# Eye morphometry, body size, and flexibility parameters in myopic adolescents

**DOI:** 10.1038/s41598-024-57347-w

**Published:** 2024-03-21

**Authors:** Kristina Kuoliene, Egle Danieliene, Janina Tutkuviene

**Affiliations:** 1 Department of Anatomy, Histology and Anthropology, Faculty of Medicine, Vilnius University, Vilnius, Lithuania; 2 Clinic of Ear, Nose, Throat and Eye Diseases, Faculty of Medicine, Vilnius University, Vilnius, Lithuania

**Keywords:** Myopia, Body size, Flexibility, Beighton score, Ocular biometry, Refractive error, Anthropometry, Anatomy, Medical research, Risk factors

## Abstract

The aim of this study was to investigate the anatomical and physiological ocular parameters in adolescents with myopia and to examine the relations between refractive error (SER), ocular biometry, body size and flexibility parameters in myopic adolescents. A cross-sectional study of 184 myopic adolescents, aged 15 to 19 years was conducted. Refractive error and corneal curvature measures of the eye were evaluated using an autorefractometer under cycloplegia. Central corneal thickness was determined by contact pachymetry. The ocular axial length, anterior and vitreous chamber depth, and lens thickness were measured using A-scan biometry ultrasonography. Height and body weight were measured according to a standardized protocol. Body mass index (BMI) was subsequently calculated. Beighton scale was used to measure joint flexibility. Body stature was positively correlated with ocular axial length (r = 0.39, *p* < 0.001) and vitreous chamber depth (r = 0.37, *p* < 0.001). There was a negative correlation between height and SER (r = − 0.46; *p* < 0.001). Beighton score and body weight had weak positive correlations with axial length and vitreous chamber depth, and a weak negative correlation with SER. A significantly more negative SER was observed in the increased joint mobility group (*p* < 0.05; U = 5065.5) as compared to normal joint mobility group: mean − 4.37 ± 1.85 D (median − 4.25; IQR − 6.25 to − 3.25 D) and mean − 3.72 ± 1.66 D (median − 3.50; IQR − 4.75 to − 2.25 D) respectively. There was a strong association between height and axial length, as well as SER. Higher degree of myopia significantly correlated with greater Beighton score (increased joint mobility).

## Introduction

Refractive errors, the most common being myopia, are the most frequent disorders in eyes^1^. The prevalence of myopia is increasing worldwide^2^, with almost five billion people predicted to be myopic by 2050^3^. Therefore, it is important to investigate all possible causes of this phenomenon, which are probably related not only to the eyes, but also to general changes in the structure and functions of the human body in space and time due to adaptation to the changing environment.

In humans, there is a positive association between ocular globe size and certain body parameters. Bigger eye size correlates with the development of myopia. Longer ocular axial length tends to be associated with myopia^4^, whereas shorter eyes appear to be hyperopic^5^.

Previous population-based studies have suggested that certain anthropometric parameters, such as height, body weight and body mass index (BMI), correlate with ocular globe dimensions and refractive error^6,7^. Since 1980, a large number of studies have investigated the relation between stature, ocular refractive error and other biometric dimensions of the eye, with inconsistent results^8^. Refractive error was found to be associated with height in several studies^9^. Likewise, taller people have been found to be more likely to have myopia in some studies^10^, yet the results are inconsistent, as other studies have found no such association^11^. Higher stature has also been found to correlate with longer axial length of the globe^12^, deeper anterior chamber, and flatter cornea, but not with the degree of myopia^13,14^. Meanwhile, greater BMI appears to be associated more with hyperopic shift in eyes^15^, however, several studies have found that taller, heavier individuals with greater BMI tend to be myopic^16^.

Biomechanical properties of the sclera have been associated with the development of refractive errors^17,18^. Stiff sclera has been found in hypermetropic and emmetropic eyes, whereas myopic eyes showed a biomechanical weakness of the scleral shell^19^. Axial elongation, the leading parameter in myopia development, is determined by the thickness, rigidity and viscoelasticity of the posterior sclera. The sclera is thinner with a loss of tissue, and scleral thinning is accompanied by a narrowing and dissociation of the collagen fibre bundles as well as a reduction in the diameter of the collagen fibrils in both experimental myopia models and human myopic eyes^20^.

The stretching of the posterior sclera is determined by genetic factors^21^ and may be associated with generalized laxity of the connective body tissue. Therefore, the relationship between body connective tissue laxity and ocular parameters, particularly the degree of myopia, may be worth investigating to search for any aetiological factors for myopia development. Connective tissue laxity can be measured using Beighton score, which is a widely used measure for generalized hypermobility or increased joint mobility^22^.

To our knowledge, no studies have been conducted to investigate potential relationships between connective tissue properties and the development of myopia in healthy adolescents using the Beighton score. In one study of individuals with joint hypermobility syndrome and Ehlers-Danlos hypermobility type, the prevalence of pathological myopia was statistically significantly higher than in the control group^23^, while a retrospective comparative study found that eyes of Marfan syndrome patients were more myopic than control eyes^24^.

Thus, the aim of our study was not only to investigate the relationship between ocular biometric parameters, the degree of myopia and anthropometric parameters, but also to evaluate joint mobility in healthy adolescents with myopia using the Beighton score, as well as to investigate the relationship between the degree of myopia, body size and the Beighton score. In addition, we aimed to find out whether factors associated with increased joint mobility may coexist with factors affecting the weak structures of the connective tissue of the eyeball and therefore may correlate with a longer axial length of the eye and the development of myopia.

## Methods

A cross-sectional study of young individuals with myopia, aged 15 to 19 years. All the teenagers were in the post-pubertal growth spurt stage, so their growth rates had slowed or nearly stopped. The study was conducted at the Eye Clinic in Vilnius, Lithuania. Ethics committee approval was obtained from the Lithuanian Bioethics Committee (Number 150000-G-225). For younger than 18-year-old participants a written, informed consent was obtained from parents, and participants provided verbal consent on the day of the examination. A written, informed consent was obtained from participants who were eighteen and older. The research adhered to the tenets of the World Medical Association’s Declaration of Helsinki.

The examination included a detailed assessment of visual acuity, identification of amblyopia and strabismus, and cycloplegia using cyclopentolate. Autorefraction, and keratometry were performed using an autorefractor (KR8800, Topcon Corp, Tokyo, Japan) after cycloplegia. Myopia was defined as spherical equivalent refractive error (SER) of < − 0.5 diopters (D), and only individuals with myopia were included in the study. The SER of each eye, measured in D was calculated with the spherical dioptric power plus half the cylindrical dioptric power. The ocular biometer (Echoscan US-4000, NIDEK, Japan) was used to measure ten valid readings of axial length and anterior chamber depth. Measures were taken on the entire cohort before the instillation of eye drops.

Our study participants (N = 184) were categorized into two groups according to the degree of myopia: (1) mild to moderate myopia (n = 143), with SER > − 6.0 D (the difference in Beighton score between mild and moderate myopia sub-groups was not statistically significant, thus we incorporated the data from the mild and moderate myopia sub-groups into one group); (2) high myopia (n = 41), with SER ≤ − 6.0 D. We chose a SER of − 6.0 D or less for high myopia because it is widely used and, if uncorrected, results in vision impairment equivalent to blindness as defined by the World Health Organization^25^.

The individual’s weight was measured by a weight beam scale. Height was measured with shoes off according to standard anthropometric methods^26^ using a metal anthropometer (Siber Hegner, made in Switzerland) and BMI was subsequently calculated (BMI = weight [in kilograms]/height^2^ [in meters]). Beighton score was used to assess an individual’s joint mobility^27,28^. The Beighton score is a modification of the Carter and Wilkinson scoring system (1964), proven to be efficient in assessing generalized joint mobility in all age groups. A nine-point scale was used, requiring study participants to perform 5 maneuvres—four passive bilateral and one active unilateral. The movements were evaluated on the right and left sides except for the movement of bending forwards (performing a trunk flexion). The maximum score for ligament laxity was nine^29^:

- one point if study participant can place their palms on the ground while bending over with the legs straight;
- one point—for each elbow that bends backwards (the presence of hyperextension);
- one point—for each knee that bends backwards; (the presence of hyperextension);
- one point—for each thumb that touches the forearm when extended backwards;
- one point—for each little finger that extends backwards beyond 90°.

The study participants were divided into two groups according to Beighton score—normal joint mobility group with Beighton score 0 to 3 and increased joint mobility group with Beighton score 4 to 9. To avoid possible bias in Beighton score, only healthy individuals with no previously diagnosed connective tissue diseases or other health disorders were included in the study.

### Statistical analysis

Statistical analysis was performed using the R software package (link https://www.R-project.org/). Right and left eye data were analyzed, but only right eye data were reported due to comparable results as spherical equivalents in the right and left eyes did not differ significantly (*p* > 0.05), therefore only a spherical equivalent value of the right eye was taken for further analysis. Shapiro–Wilk test revealed that continuous variables were not normally distributed. Mann–Whitney U tests were calculated to determine the association of demographic variables (age and biological sex) and body size parameters (height, weight, BMI), also Beighton score with ocular biometric values.

Chi-squared test (χ^2^) was used to test the independence of qualitative characteristics—gender differences between normal and increased joint flexibility groups (Beighton score grade), and between mild to moderate and high myopia groups. Kruskal–Wallis test with Dunn’s post hoc test was applied to compare body size and flexibility parameters among the sub-groups of mild, moderate, and high myopia groups. Simple linear regression was performed to evaluate the effect of height, weight, BMI, Beighton score value on the biometric indices and SER. Statistical significance was maintained at a *p*-value less than 0.05.

Correlations of both ocular biometric parameters and SER with both body size measures and Beighton score were calculated using Spearman’s correlation coefficient (r).

Multiple linear regression models were constructed to evaluate the influence of height, weight, BMI, Beighton scale, and biological sex on SER and ocular biometric parameters including axial length, anterior chamber depth, and corneal curvatures.

## Results

The study included 184 adolescents with myopia between the age of 15 and 19 years. There were more females—105 individuals (57.07%)—than males in our study, this difference was not statistically significant (*p* > 0.05, U = 4378.0). The mean age of the study subjects was 17.33 ± 1.17 years. Mean age was very similar in females (17.38 ± 1.16 years; median 17; interquartile range (IQR) 16–18 years) and in males (17.38 ± 1.18 years; median 17; IQR 16–18 years). Body size characteristics and Beighton score of the study population are shown in Table 1.

**Table 1:** Age, body size indices and Beighton score in boys and girls with myopia.

Variable	Total (n = 184; 100%)			Girls (n = 105; 57.07%)			Boys (n = 79; 42.93%)		
	Mean (SD)	Range (min–max)	Median (IQR)	Mean (SD)	Range (min–max)	Median (IQR)	Mean (SD)	Range (min–max)	Median (IQR)
Age (years)	17.33 (1.77)	15–19	17 (16–18)	17.38 (1.16)	15–19	17 (16–18)	17.27 (1.16)	15–19	17 (16–18)
Height (cm)	176.9 (10.02)	156.0–196.0	176 (169–185)	171.9 (7.14)	156.0–189.0	172* (167–178)	183.46 (9.52)	159.0–196.0	186* (178–190)
Weight (kg)	64.96 (11.83)	45.3–98.2	63 (56.6–75)	58.93 (7.36)	45.3–85.0	57.2* (54.2–62.3)	75.31 (10.06)	56.2–98.2	75.8* (67.9–82.4)
BMI (kg/m^2^)	20.95 (2.29)	16.79–28.4	20.86 (19.21–22.31)	19.91 (1.81)	16.79–28.4	19.47* (18.72–20.91)	22.34 (2.12)	16.8–28.38	22.28* (21.36–23.49)
Beighton score	3.10 (1.86)	0–8	3 (2–4)	3.01 (1.63)	0–7	3 (2–4)	3.24 (2.14)	0–8	3 (1–4)

*p < 0.001 (Mann–Whitney U test): differences between biological sexes; BMI: body mass index; SD: standard deviation; IQR: interquartile range.

Obviously, males were significantly taller and had bigger body mass index when compared to females (*p* < 0.001, U = 1430.5). There were no significant differences in ocular parameters between biological sexes, except for corneal curvature, as men had steeper corneas (average 7.94 ± 0.21; median 7.95; IQR 7.81–8.06) compared to women (average 7.87 ± 0.23; median 7.83; IQR 7.72–8.05) (*p* < 0.05, U = 3414.0).

Refractive error was expressed as a SER. There were no significant differences in ocular SER parameters between male and female study subjects–average SER was − 4.06 ± 1.94 D (median − 4.25; IQR − 5.50 to − 2.38 D) in men and − 4.01 ± 1.65D in women (median − 4.0; IQR − 5.25 to − 3.0 D) (*p* > 0.05, U = 4239.0).

High myopia was observed in 22.3% of the study subjects, 77.7% of individuals had mild to moderate myopia.

Kruskal–Wallis test showed a statistically significant difference for height (H = 30.74; p < 0.001), weight (H = 17.36; *p* < 0.001), BMI (H = 10.49; *p* < 0.01) and Beighton score (H = 6.48; *p* < 0.05) between all three myopia groups. Dunn’s post-hoc test revealed no significant pairwise difference (*p* > 0.05) in Beighton score between the mild and moderate myopia groups (Table 2), but the Beighton score was clearly higher in the high myopia group (*p* < 0.05). There was a clear trend (Table 2) that height was greater with increasing degree of myopia, and the difference in height between all groups was highly statistically significant (*p* < 0.001). This trend was not observed in BMI, as a Dunn’s post-hoc test revealed a statistically significant difference in BMI only between subjects with moderate and high myopia (*p* < 0.01).

**Table 2:** Body size and flexibility parameters in boys and girls according to the degree of myopia.

Body size and flexibility parameters	Mild myopia (n = 46; 25.0%)	Moderate myopia (n = 97; 53.71%)	High myopia (n = 41; 22.28%)	H value	*p* value
Height (mean ± SD, cm)	170.54 ± 8.34	177.54 ± 9.31	182.34 ± 9.78	30.74	< 0.001
Height (median (IQR), cm)	169.5 (165–176)	176 (170–186)	180 (177–191)		
Weight (mean ± SD, kg)	61.94 ± 9.69	64.90 ± 11.04	72.99 ± 13.07	17.36	< 0.001
Weight (median (IQR), kg)	60.30 (54.88–68.88)	61 (56.30–76.0)	70.70 (63.60–85.0)		
BMI (mean ± SD, kg/m^2^)	21.22 ± 2.37	20.48 ± 2.17	21.77 ± 2.23	10.49	< 0.01
BMI (median (IQR), kg/m^2^)	20.90 (19.39–22.62)	20.06 (18.81–22.10)	21.85 (20.10–22.70)		
Beighton score (Mean ± SD)	2.93 ± 1.65	2.90 ± 1.78	3.80 ± 2.15	6.48	< 0.05
Beighton score (median (IQR)	3 (2–4)	3 (1–4)	4 (2–5 )		

BMI: body mass index; SD: standard deviation; IQR: interquartile range; cm: centimeters; kg: kilograms; m: meters; n: number of subjects.

**Figure 1:**
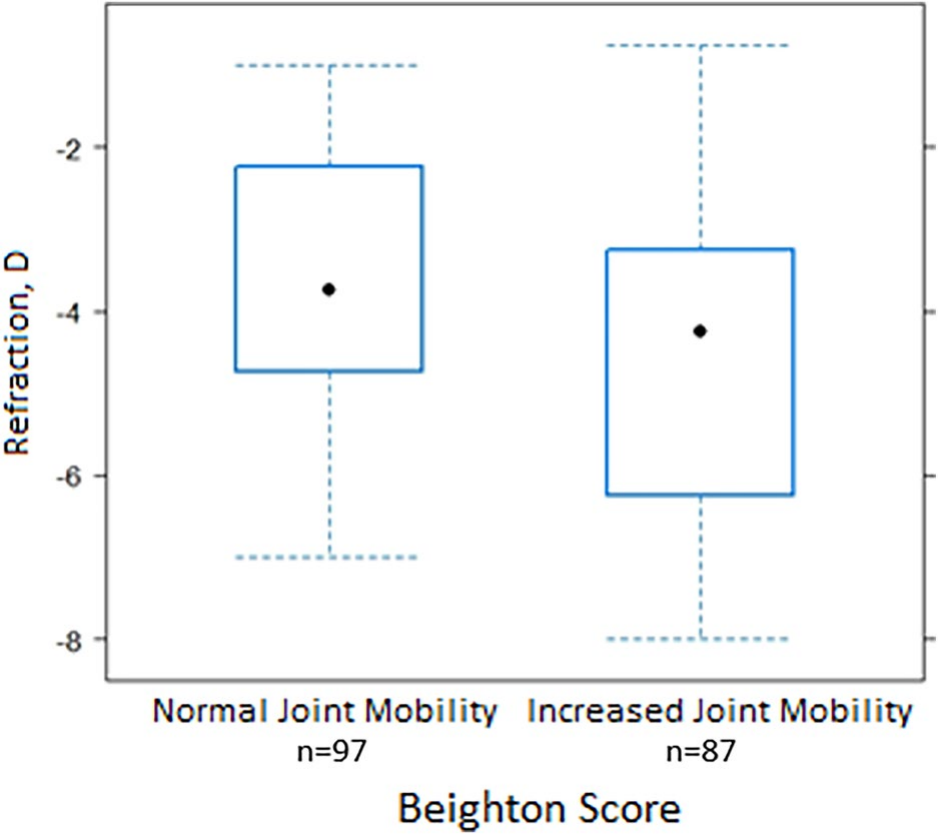
Boxplot of SER and Beighton Score.

In addition, the study participants were divided into two groups according to Beighton score. There were 87 (47.28%) individuals in the increased joint mobility group. Although increased joint mobility was more frequent in females (56.98%) than in males (43.02%), this difference was not statistically significant between biological sexes (*p* > 0.05). As presented in Fig. 1 and Table 3, a significantly more negative SER was observed in the increased joint mobility group as compared to the normal joint mobility group: mean − 4.37 ± 1.85 D (median − 4.25; IQR − 6.25 to − 3.25 D) and mean − 3.72 ± 1.66 D (median − 3.50; IQR − 4.75 to − 2.25 D) respectively, *p* < 0.05; U = 5065.5. Statistically significantly higher degree of myopia, longer axial length, longer vitreous chamber, and greater central corneal thickness were observed in the increased joint mobility group (*p* < 0.05, Mann–Whitney U test).

**Table 3:** Statistical data for the Boxplot of SER and Beighton Score.

Joint mobility type	Descriptive statistics for SER for two groups of joint mobility						
	Min	Q1	Median	Average	SD	Q3	Max
Normal (Beighton score < 4; n = 97; 52.72%)	− 7	− 4.75	− 3.50*	− 3.72	1.66	− 2.25	− 0.75
Increased (Beighton score ≥ 4; n = 87; 47.28%)	− 8	− 6.25	− 4.25*	− 4.37	1.85	− 3.25	− 0.75

SD, standard deviation; Q1, first quartile; Q3, third quartile; Min, minimum; Max, maximum; *p < 0.05; U = 5065.5.

Significant values are in bold.

Univariate logistic regression showed that higher stature and bigger weight, also BMI as well as greater joint mobility were associated with high degree myopia. Individuals with longer axial length and vitreous chamber length as well as greater central corneal thickness had an increased risk for developing high degree of myopia. Odds ratios for high degree of myopia according to ocular and body size parameters in the study subjects are shown in Table 4.

**Table 4:** Odds ratios for high degree of myopia in relation to variables.

Variable	*p* value	OR (odds ratio)	95% CI
Gender	0.617	1.19	(0.59–2.40)
Age, years	0.263	1.19	(0.88–1.61)
Height, cm	**0.000**	1.08**	**(1.04–1.12)**
Weight, kg	**0.000**	1.07**	**(1.03**–**1.10)**
BMI, cm/kg^2^	**0.010**	1.22*	**(1.05**–**1.42)**
Beighton score	**0.008**	1.30**	**(1.08**–**1.59)**
Axial length, mm	**0.000**	6.26**	**(3.55–11.18)**
Anterior chamber depth, mm	0.649	0.70	(0.15–3.39)
Lens thickness, mm	0.265	2.638	(0.47–14.39)
Vitreous chamber length, mm	**0.000**	6.75**	**(3.93–12.92)**
Central corneal thickness, µm	**0.006**	0.98**	**(0.96–0.99)**
Corneal curvature, D	0.483	1.10	(0.83–1.46)
Basic corneal curvature, mm	0.101	3.97	(0.80–21.86)

*p < 0.05; **p < 0.01; BMI, body mass index.

Significant values are in bold.

Bivariate correlation analysis of height, weight, BMI and Beighton score with SER as well as ocular biometrics are presented in Table 5. These correlations were of low to moderate strength. Body stature was positively correlated with axial length (r = 0.39, *p* < 0.001) and vitreous chamber depth (r = 0.37, *p* < 0.001).

**Table 5:** Bivariate correlations (Spearman’s correlation coefficients) of body stature, weight, body mass index and Beighton score with SER and ocular biometric parameters.

	Height (cm)	Weight (kg)	BMI (kg/m^2^)	Beighton score
SER (D)	− 0.46*	− 0.22*	− 0.09*	− 0.13*
AL (mm)	0.39*	0.20*	0.07	0.13*
ACD (mm)	− 0.11	− 0.09	− 0.06	− 0.06
LT (mm)	0.08	− 0.01	− 0.04	− 0.10
VCD (mm)	0.37*	0.21*	0.11	0.16*
CCT (µm)	− 0.08	0.01	− 0.01	0.14*
CC (mm)	0.13*	0.14	− 0.11	− 0.04

SER, spheric equivalent refractive error; AL, axial length; ACD, anterior chamber depth; LT, lens thickness; VCD, vitreous chamber depth; CCT, central corneal thickness; CC, corneal curvature; BMI, body mass index; *p < 0.05.

There was a negative correlation between height and SER (r = − 0.46; *p* < 0.001). Beighton score and body weight had weak positive correlations with axial length and vitreous chamber depth, and a weak negative correlation with SER.

As shown in Table 6, the dependent variable in a separate regression model was ocular biometrics and SER, body size indicators were independent variable adjusted for other covariates. In the final model of this table, a 10-cm taller person, after controlling for age in years, biological sex, weight could be expected to have a 0.56-mm (*p* < 0.001) increase in axial length and a 1.26 D (*p* < 0.001) decrease in SER, resulting in higher myopia.

**Table 6:** Linear regression models of ocular biometry and SER by height, weight, BMI, and Beighton score.

	Crude data	Adjusted for age and biological sex	Adjusted for age, biological sex and weight	R^2^ final models
Height (10 cm)				
AL (mm)	0.39 (0.25; 0.53)***	0.48 (0.32; 0.66)***	0.56 (0.34; 0.78)***	0.16
ACD (mm)	− 0.02 (− 0.06; 0.008)	− 0.03 (− 0.007; 0.008)	− 0.03 (− 0.08; 0.02)	–
LT (mm)	0.01 (− 0.02; 0.04)	0.004 (− 0.03; 0.04)	0.03 (− 0.01; 0.08)	–
VCD (mm)	0.38 (0.024; 0.052)***	0.48 (0.31; 0.65)***	0.49 (0.27; 0.72)***	0.155
CCT (µm)	− 1.108 (− 4.28; 2.13)	− 3.98 (− 7.84; − 0.13)*	− 3.92 (− 8.98; 1.13)	0.034
CC (mm)	0.03 (− 0.007; 0.059)	0.008 (− 0.03; 0.05)	0.04 (− 0.01; 0.09)	–
SER (D)	− 0.8 (− 1.05; − 0.59)***	− 1.19 (− 1.45; − 0.92)***	− 1.26 (− 1.61; − 0.92)***	0.309
Weight (10 kg)				
AL (mm)	0.25 (0.13; 0.37)***	0.36 (0.19; 0.53)***	− 0.07 (− 0.15; 0.29)	0.163
ACD (mm)	− 0.02 (− 0.05; 0.009)	− 0.03 (− 0.07; 0.011)	− 0.01 (− 0.06; 0.04)	–
LT (mm)	0.00 (− 0.02; 0.026)	− 0.02 (− 0.05; 0.02)	− 0.04 (− 0.08; 0.01)	–
VCD (mm)	0.27 (0.09; 0.356)***	0.41 (0.24; 0.58)***	− 0.17 (− 0.05; 0.40)	0.167
CCT (µm)	0.11 (− 2.44; 3.41)	− 3.41 (− 7.12; 0.31)	− 1.48 (− 6.57; 3.60)	–
CC (mm)	0.01 (− 0.018; 0.041)	− 0.02 (− 0.06; 0.02)	− 0.04 (− 0.09; 0.008)	–
SER (D)	− 0.53 (− 0.74; − 0.33)***	− 0.95 (− 1.23; − 0.69)***	− 0.33 (− 0.68; 0.008)^	0.326
BMI (kg/m^2^)				
AL (mm)	− 0.28 (− 0.38; 0.94)	− 0.02 (− 0.76; − 0.81)	− 4.04 (− 9.74; 1.66)	–
ACD (mm)	− 0.037 (− 0,17; 0,01)	0.03 (− 0.19; 0.14)	− 0.1 (− 1.43; 1.22)	–
LT (mm)	− 0.05 (− 0.02; 0.076)	− 0.12 (− 0.27; 0.03)	− 0.47 (− 1.65; 0.70)	–
VCD (mm)	− 0.5 (− 0.15; 1.18)	− 0.37 (− 0.41; 1.16)	− 3.39 (− 9.13; 2.34)	–
CCT (µm)	5.55 (− 8.48; 19.58)	− 1.52 (− 18.05; 15.00)	130.00 (1.45; 250.86)	0.038
CC (mm)	− 0.01 (− 0.16; 0.132)	− 0.13 (− 0.29; 0,04)	0.69 (− 0.62; 2.02)	–
SER (D)	− 0.56 (− 1.69; 0.56)	− 0.58 (1.92; 0.74)	8.99 (0.32; 17.66)	0.338
Beighton score				
AL (mm)	0.81 (− 0.006; 1.62)^	0.8 (− 0.05; 1.56)	0.50 (− 0.25; 1.26)	0.169
ACD (mm)	− 0.09 (− 0.27; 0.08)	− 0.08 (− 0.26; 0.09)	− 0.06 (− 2.24; 0.11)	–
LT (mm)	− 0.06 (− 0.21; 0.096)	− 0.06 (− 0.2; 0.09)	− 0.05 (− 0.21; 0.11)	–
VCD (mm)	1.01 (0.204; 1.82)*	0.96 (0.15; 1.76)^	0.67 (− 0.08; 1.43)	0.173
CCT (µm)	15.922 (− 1.88; 32.33)	13.87 (− 3.22; 30.97)	16.79 (− 0.34; 33.94)^	0.053
CC (mm)	− 0.06 (− 0.23; 0.12)	− 0.07 (− 0.25; 0.10)	− 0.05 (− 0.24; 0.12)	0.026
SER (D)	− 1.63 (− 3.00; − 0.26)*	− 1.56 (− 2.94; 0.19)^	− 0.88 (2.04; 0.28)	0.321

Each value represents a separate regression model, with the ocular biometric parameters and SER as a dependent variable and anthropometric values as the independent variable adjusting for other covariates. Models for height are adjusted for weight and vice versa. Data represent the 95% confidence interval: SER, spheric equivalent refractive error; AL, axial length; ACD, anterior chamber depth; LT, lens thickness; VCD, vitreous chamber depth; CCT, central corneal thickness; CC, corneal curvature; BMI, body mass index; ***p < 0.001, **p = 0.001, *p = 0.01, ^p < 0.05.

An increase in Beighton score can result in significant increase in AL (+ 0.81 mm, *p* < 0.05), VCD (+ 1.1 mm, *p* < 0.05), and a decrease in SER (− 1.63, *p* < 0.05) when adjusted for age and biological sex.

## Discussion

Earlier studies have established that longer axial length is a major ocular biometric factor associated with myopia^30^. Some studies have found a positive correlation between ocular axial length and stature^31,32^. A tendency has been found that taller and heavier individuals are more myopic and shorter stature is associated more with emmetropia or hyperopia^33,34^. These findings are inconclusive and inconsistent, since several population-based studies analyzed the relation between body stature and refractive error and found no significant association^35^.

In the present study, of course, there were significant differences in body size parameters between biological sexes—males being significantly taller and with bigger weight when compared to females. However, there were no significant differences in ocular parameters between biological sexes, except for corneal curvature, as women had flatter corneas compared to men. This may be because there was no significant difference in refractive status between male and female individuals in our study. Thus, we focused on the degree of myopia regardless of biological sex. We examined myopic adolescents and compared their body size indices with ocular biometry and SER for groups of high, moderate, and low myopia.

Our study results are consistent with previous studies, where height, BMI, and axial length, as well as refractive error correlate in both male and female myopic adolescents: high myopia was linked to bigger BMI, taller height, longer ocular axial length, longer vitreous chamber. Earlier studies have provided evidence of a strong correlation between myopia and greater height both in adults and in children^36^. Saw et al. found a strong relationship between height and axial length in Chinese school children aged 7 to 9 years and found that taller children tended to have myopic refractive error^37^. In a large population-based study of Australian children, Ojaimi et al. found a strong association between height and axial length, and corneal radius, but not refractive error^38^. These discrepancies between the results of these studies could be due to varying design and sample sizes, different age ranges, and refractive error measurement techniques.

In previous population-based studies weight and BMI were associated with hyperopia^37^. As mentioned before, we only studied individuals with myopia and found that subjects in the high myopia group tended to be heavier and with greater BMI. Cordain et al., in their study related to evolutionary aspects of the aetiology and pathogenesis of juvenile-onset myopia, suggested that myopes are typically taller and heavier and have higher BMI because of changing dietary patterns^39^. This has also been shown by Terasaki et al. in their recent study of Japanese elementary school children in which a strong association has been found between myopia, bigger body weight, higher BMI, and westernized dietary habits^40^. This supports the hypothesis that an increase in myopia worldwide might be related to an environmentally driven increase in axial length in relation to general body size changes^31^.

As there is strong evidence that body height and BMI have increased over the past few decades^41,42^, these changes in body size may be associated with increased axial length and myopia. These findings suggest that ocular growth at a time when body stature is also increasing may have a shared mechanism of action. In our study, all the children studied were in the post-puberty growth spurt stage, so the possible mechanism of the effect of growth on the eye parameters and vision has already occurred.

Earlier studies have linked increased scleral matrix remodeling to biomechanical weakening of the sclera that leads to excessive elongation of the ocular globe and the development of myopia^43^, and scleral thickness has been found to decrease with increasing ocular axial length^44^. Several changes in scleral composition, biomechanics and structure have been identified in human myopia and experimental animal myopia. Both posterior and anterior sclera was found to be thinner in myopic eyes, especially in high myopia individuals^45,46^.

Therefore, in addition to providing comparison between body size to ocular biometric parameters and refractive error in a sample of adolescents with myopia, our study also included comparison of ocular parameters with generalized joint mobility using Beighton scale. To the best of our knowledge, there is no other study published that examined associations between ocular parameters in myopic individuals and connective tissue parameters to date. We found that individuals in the high myopia group had a higher value of Beighton score and, thus, increased generalized connective tissue flexibility.

We hypothesize that this could primarily be related to the phenomenon of connective tissue insufficiency in accelerated populations, because the development of connective tissue and its energy costs are evolutionarily very expensive and cannot be unlimited. It is highly likely that the lack of connective tissue is then compensated for by more abundant body mass (as support or bracing), as the individuals in our study had a higher BMI^47–49^.

There is a general trend in human evolution during the last 4 million years that body mass and stature increases over time, with an even bigger relative increase in brain size. These changes are related to hypothesized environmental, demographic, dietary, social, and technological factors^50^. The above-mentioned evolutionary changes are associated with an increasing incidence in certain physiological and pathological conditions and morbidity. The prevalence of myopia has increased over time and the rate continues to grow further over time^51,52^. In addition, there is an increase in connective tissue related conditions, such as scoliosis and spine deformities both in children and adults as well as the incidence of spinal disc herniations^53–55^. The rate of abdominal wall hernia repair has been reported to increase over time^56^.

Variation of the results of population-based myopia studies could mean that other factors that affect height and refractive error separately exist. Socioeconomic status, education, and diet have been associated with greater height and increased risk for myopia development. We examined another independent factor that may be related to stature and refractive status—generalized connective tissue laxity assessed using the Beighton scale.

In our study, general connective tissue weakness determined by Beighton score was associated with development of higher degree myopia (but not with moderate and mild myopia), and the average myopic refractive error was greater in the increased joint mobility group. Our study suggests that general connective tissue weakness should be investigated further to find any possible associations with changes in scleral composition and scleral remodeling that leads to elongation of the globe and the development of myopia.

Molecular studies of the human connective tissue extracellular matrix composition have established changes in the amount of collagen and elastin related to different degenerative diseases, such as scoliosis, spinal disc degeneration, and general connective tissue laxity^57^. Genetic studies of the connective tissue suggest that increased connective tissue laxity may be not an isolated condition but a certain form or disorder arising from disruption and changes in the collagen composition under certain genetic circumstances^58,59^. We hypothesize that certain indicators and markers need to be found, which may be shared among systems for general changes in body composition over time due to environmental, dietary, socioeconomical, and technological changes^60^.

In conclusion, in our study there was a strong association between height and axial length, as well as spherical equivalent refractive error. Tall height, weight, and BMI, as well as increased joint mobility and total connective tissue laxity (as determined by the Beighton score) were significantly correlated with a high degree of myopia. In addition, individuals in the high myopia group had longer axial length and vitreous chamber length, as well as greater central corneal thickness.

## Data Availability

The datasets used and/or analyzed during the current study available from the corresponding author on a reasonable request.
